# A Homoploid Hybrid Between Wild *Vigna* Species Found in a Limestone Karst

**DOI:** 10.3389/fpls.2015.01050

**Published:** 2015-12-01

**Authors:** Yu Takahashi, Kohtaro Iseki, Kumiko Kitazawa, Chiaki Muto, Prakit Somta, Kenji Irie, Ken Naito, Norihiko Tomooka

**Affiliations:** ^1^Genetic Resources Center, National Institute of Agrobiological SciencesTsukuba, Japan; ^2^Department of International Agricultural Development, Tokyo University of AgricultureTokyo, Japan; ^3^Department of Agronomy, Faculty of Agriculture at Kamphaeng Saen, Kasetsart UniversityNakhon Pathom, Thailand

**Keywords:** genus *Vigna*, wild crop relatives, genetic resource, drought tolerance, stress tolerance, homoploid hybrid speciation, introgressive hybridization, natural hybrid

## Abstract

Genus *Vigna* comprise several domesticated species including cowpea and mungbean, and diverse wild species. We found an introgressive hybrid population derived from two wild species, *Vigna umbellata* and *Vigna exilis*, in Ratchaburi district, Thailand. The hybrid was morphologically similar to *V. umbellata* but habituated in a limestone rock mountain, which is usually dominated by *V. exilis*. Analyzing simple sequence repeat loci indicated the hybrid has undergone at least one round of backcross by *V. umbellata*. We found the hybrid acquired vigorous growth from *V. umbellata* and drought tolerance plus early flowering from *V. exilis*, and thus has taken over some habitats of *V. exilis* in limestone karsts. Given the wide crossability of *V. umbellata*, the hybrid can be a valuable genetic resource to improve drought tolerance of some domesticated species.

## Introduction

Natural hybrid between species is considered to drive adaptation and speciation (Strasburg et al., 2012). However, in plants, interspecific outcrossing is expected to frequently occur but is rarely discovered, since just one round of backcross by either of the parent may generate plants that greatly resemble the species to which they were backcrossed (Anderson, 1948). If the second backcross occurs, it becomes almost impossible to distinguish the hybrid from the progenitors (Anderson, 1936). As such, despite its hypothetical importance, the reported hybrid species have been limited and are mainly alloploids, which is reproductively isolated from their diploid progenitors and generate new lineages (Paun et al., 2009). In contrast, homoploid hybridization is much more difficult to detect and thus fewer cases have been identified (Paun et al., 2009). It has been proposed that homoploid hybrids can acquire novel combinations of traits or transgressive phenotypes, with which they can colonize into ecological niches that are inaccessible to both of the parental species (Arnold et al., 2012). To date, however, such “adaptive” traits have been identified only in three cases; *Helianthus*, (Rieseberg et al., 2003), *Pinus* (Mao and Wang, 2011), and *Iris* (Arnold et al., 2012).

Genus *Vigna* comprise more than 100 species (Tomooka et al., 2002; Maxted et al., 2004) including several domesticated species such as cowpea [*Vigna unguiculata* (L.) Walp.], mungbean [*Vigna radiata* (L.) R. Wilczek], and azuki bean [*Vigna angularis* (Willd.) Ohwi and Ohashi]. Since wild species inhabit various environments such as marine beach, deserts and swamps, *Vigna* species are now considered as a valuable genetic resource for stress tolerance (Chankaew et al., 2014a; Tomooka et al., 2014).

**Figure 1:**
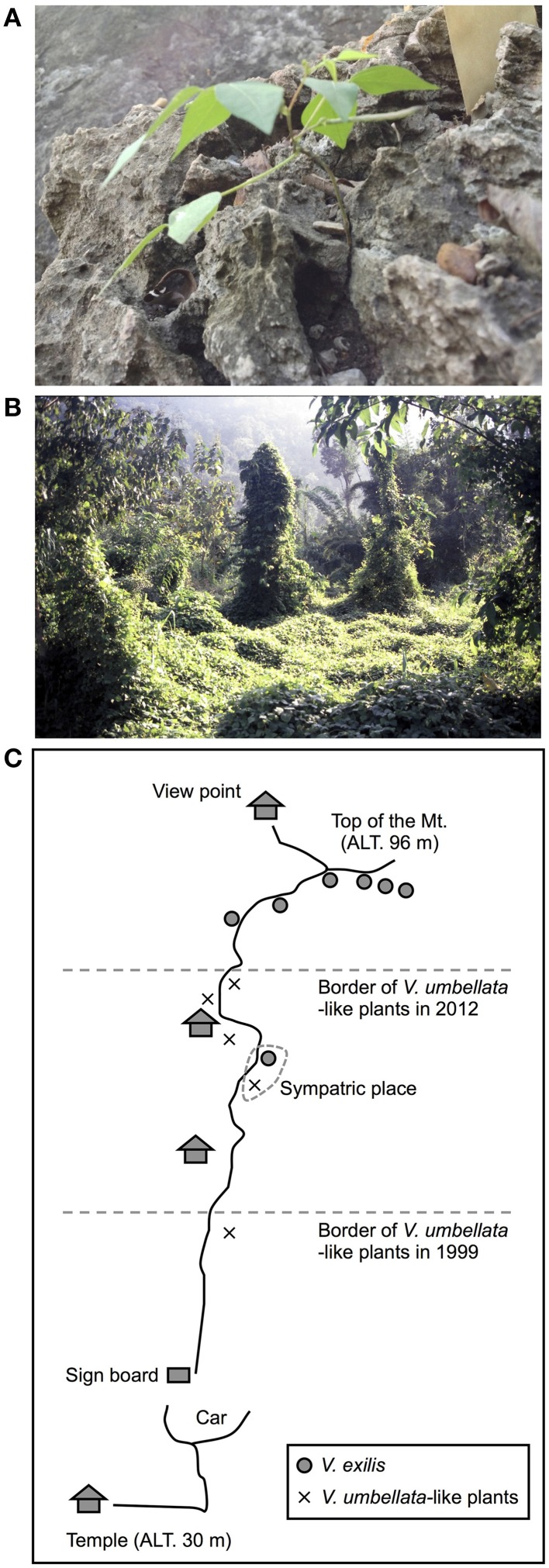
**Photos of *V. exilis* (A) and *V. umbellata* (B) and a schematic map a limestone rock mountain near Wat Rat Singkhon (C)**.

One of the particular interests is *Vigna exilis* Tateishi and Maxted, which grows on limestone rocks (Tomooka et al., 2002). There extends limestone karst landscape in north and west Thailand (Clemens et al., 2006), and *V. exilis* has been found only in the mogote-like hills or mountains in this region. It grows its root into cracks of the rocks in open or slightly shaded habitats (Tomooka et al., 2002; Figure 1A). Since limestone is mainly composed of calcium carbonate (CaCO3), this species is expected as a valuable genetic resource for alkaline tolerance (Tomooka et al., 2014). It used to be described as an accession of *Vigna dalzelliana* (O. Kuntze) Verdcourt but was recently admitted as an independent species (Tateishi and Maxted, 2002). The distinguishing characteristics are its flat, thin and linear seed shape and flat style beak (Tomooka et al., 2002).

*V. exilis* is allopatric but mutually exclusive with another species *Vigna umbellata* (Thunb.) Ohwi and Ohashi, which is the progenitor of the cultivated rice bean (Tomooka et al., 2002). The wild form of *V. umbellata* is often found in mountain ranges in India through the Southeast and East Asia, as well as disturbed environments such as roadside and cultivated fields. It is self-pollinating but has relatively higher rate of outcross, which is estimated to be 13.3–41.6% (Das and Dana, 1987). The most outstanding feature of this species is its vigorous growth (Figure 1B).

Although *V. exilis* and *V. umbellata* are genetically close to each other, they are distinctive species and no evidence of gene flows have been found between them (Tian et al., 2013).

In this study, we first explored limestone karsts in west Thailand to collect *V. exilis* in 1999. Besides *V. exilis*, we also found a population that looked like *V. umbellata* at the foot of a limestone mountain. We identified it as *V. umbellata* based on its seed morphology, however, in 2012 we found the *V. umbellata*-like plants had expanded upwards of the mountain. Thus, we suspected that the population derives from hybridization between *V. umbellata* and *V. exilis*, and performed morphological and molecular analyses, followed by evaluation for tolerance to some abiotic stresses. The study also revealed the value of wild genetic resources for improving stress tolerance in crops.

## Materials and methods

### Exploration

We conducted exploration in west Thailand in November 18th, 1999 and November 13th, 2012 as a part of the program in National Institute of Agrobiological Sciences (NIAS) gene bank. We visited a limestone rock mountain near Wat Rat Singkhon in Ratchaburi district (13°34′29″N, 99°46′29″E). When we found *Vigna* population, bulk seeds were collected from naturally growing plants. Identification of the *Vigna* species was based on a taxonomic key (Tomooka et al., 2002). The latitude, longitude and altitude were measured by Garmin GPSmap 60CSx using WGS84 world geodetic system. As a passport data, latitude, longitude, altitude, characteristics of the collection site and plant population were recorded.

### Plant materials

We refer the accessions of *V. umbellata*-like plants as “unidentified.” In addition to the three unidentified accessions, we tested three accessions of *V. exilis*, 13 accessions of *V. umbellata*, and four accessions of *V. dalzelliana* (Table 1). The unidentified accessions included the one we collected in Ratchaburi district in Thailand in 1999 (JP210644), and the ones we collected at the same location in 2012 (JP247174 and JP247175). *V. umbellata* accessions included four wild accessions and one escape accession (an escape of a cultigen) that we collected in Thailand in 1999, one wild accession collected in Cambodia, and seven cultivated accessions (Table 1). The two accessions of *V. exilis* were collected in Thailand in 2012. The four accessions of *V. dalzelliana* were collected in Myanmar in 2012.

**Table 1:** **A list of plant materials**.

**Symbol**	**Species**	**Status**	**Origin**	**Accession No**.	**Purpose**	**Year collected**
uni1	Unidentified	Wild	Thailand	JP210644	Phylogeny, crossing, ploidy, stress test	1999
uni2	Unidentified	Wild	Thailand	JP247174	Phylogeny, ploidy	2012
uni3	Unidentified	Wild	Thailand	JP247175	Phylogeny, ploidy	2012
umw1	*V. umbellata*	Wild	Thailand	JP207982	Phylogeny, crossing, ploidy, stress test	1999
umw2	*V. umbellata*	Wild	Thailand	JP210639	Phylogeny, ploidy	1999
umw3	*V. umbellata*	Wild	Thailand	JP210642	Phylogeny, ploidy	1999
umw4	*V. umbellata*	Wild	Thailand	JP210676	Phylogeny, ploidy	1999
umw5	*V. umbellata*	Wild	Cambodia	JP251332	Crossing, ploidy	2013
umc1	*V. umbellata*	Cultivated	Myanmar	JP212059	Phylogeny	2001
umc2	*V. umbellata*	Cultivated	Myanmar	JP217439	Phylogeny	2002
umc3	*V. umbellata*	Cultivated	Nepal	JP223027	Phylogeny	1984
umc4	*V. umbellata*	Cultivated	Nepal	JP223046	Phylogeny	1984
umc5	*V. umbellata*	Cultivated	India	JP225373	Phylogeny	Unknown
umc6	*V. umbellata*	Cultivated	India	JP239864	Phylogeny	Unknown
umc7	*V. umbellata*	Cultivated	China	JP227454	Phylogeny	Unknown
ume1	*V. umbellata*	Escape	Thailand	JP210647	Phylogeny	1999
exi1	*V. exilis*	Wild	Thailand	JP247172	Phylogeny, ploidy, stress test	2012
exi2	*V. exilis*	Wild	Thailand	JP247173	Phylogeny, ploidy	2012
exi3	*V. exilis*	Wild	Thailand	JP205884	Crossing, ploidy	1999
dal1	*V. dalzelliana*	Wild	Myanmar	JP210812	Phylogeny	2001
dal2	*V. dalzelliana*	Wild	Myanmar	JP210813	Phylogeny	2001
dal3	*V. dalzelliana*	Wild	Myanmar	JP210815	Phylogeny	2001
dal4	*V. dalzelliana*	Wild	Myanmar	JP210816	Phylogeny	2001

For cross-compatibility test, we planted one unidentified accession (JP210644), two wild accessions of *V. umbellata* (JP207982 and JP251332), and one accession of *V. exilis* (JP205884; Table 1).

For stress tolerance evaluation, we tested an unidentified accession (JP210644), a wild *V. umbellata* accession (JP207982), and a *V. exilis* accession (JP247172; Table 1).

### DNA sequencing

We extracted DNA from young leaves of 21 accessions (Table 1) for phylogenetic studies, using CTAB method (Murray and Thompson, 1980).

We sequenced *atpB-rbcL* spacer region in chloroplast genome and the rDNA internal transcribed spacer (ITS) region in nucleus genome of 21 accessions (Table 1). We used the same primer sets as Doi et al. (2002). We performed PCR using KOD-Plus-Neo 1 unit (TOYOBO) following manufacturer's protocol, using GeneAmp PCR system 9700 (Applied Biosystems). The PCR cycle was as follows: 94°C for 2 min, 35 cycles of 98°C for 10 s, and 68°C for 1 min. We directly sequenced the amplified products using BigDye Terminator v3.1 Cycle Sequencing Kit (Applied Biosystems) using an ABI Model 3130 (Applied Biosystems) according to the manufacturer's protocol.

We deposited all the sequence information to DNA Data Bank of Japan (www.ddbj.nig.ac.jp). Sequence IDs are listed in Supplementary Table 1.

### Phylogenetic analysis

We analyzed the sequence data using MEGA6 (Tamura et al., 2013). We first aligned all the sequences to each other using Clustal W (Thompson et al., 1994), and trimmed unalignable sequences. We then drew phylogenetic tree by neighbor-joining method (Saitou and Nei, 1987) and examined significance level by bootstrap test with 1000 repeats.

### SSR analysis

We tested 24 SSR markers that we observed successful amplification in *V. exilis* in our previous study (Chankaew et al., 2014b). All primers were labeled with 6-FAM, HEX, or NED (Applied Biosystems). We performed PCR with Multiplex PCR Master Mix (Qiagen) following the manufacturer's protocol, and fragment analysis using ABI PRISM 3130xl DNA Analyzer (Applied Biosystems). Genotypes were determined by GeneMapper 4.0 (Applied Biosystems). We calculated genetic distance using Populations 1.2.30 (http://bioinformatics.org/~tryphon/populations/), and constructed phylogenetic tree by neighbor-joining method (Saitou and Nei, 1987). We also performed bootstrap test with 1000 repeats.

### Ploidy level determination

Ploidy level was analyzed using a CyFlow PA (Partec). The leaves were chopped with a razor blade in a Petri dish containing the nuclei extraction buffer (solution A of the High Resolution Kit for Plant DNA, Partec). After filtration through a 30 μm nylon sieve, a staining solution of dye 4,6-diamidino-2-phenylindole-2HCl (solution B of the Kit) was added. The nuclei mixture was analyzed using the CyFlow PA. DNA content in each nucleus was evaluated using the Partec software package.

### Evaluation of alkaline tolerance

Alkaline tolerance was evaluated using the soil adjusted to different pH in a green house in July 2013. Granular culture soil with pH6.0 was used for control experiment, and was adjusted to pH8, pH10, or pH12 using slacked lime (Calcium hydroxide). Soil pH was determined using a pH meter (LAQUAtwin B-711, HORIBA) in 1:5 fine grinding soil:water suspension. Five seeds for each accession were sown in a plastic pot of 10 cm height and 6.5 cm diameter containing the soil. We prepared two pots for each experiment as replications. After 14 days from sowing, the plant was divided into the above-ground part and the root, and they were dried at 80°C for 2 days in an oven. The relative ratio of dry weight per plant was calculated against the control experiment.

### Evaluation of drought tolerance

Drought tolerance was evaluated for the plants grown in growth chamber (TGC-700, ESPEC MIC corp.). The growth conditions were 1500 μmol m^−2^ s^−1^ of photosynthetic photon flux density, 60% relative humidity, 12 h photoperiod and day/night temperature of 30/25°C. Three seeds for each accession were sown in a plastic pot of 10 cm height and 6.5 cm diameter containing granular culture soil of high water permeability. We prepared five pots for one accession as replications. For water application, all pots were soaked into water of 1 cm depth up to the drought onset. At the time when the first leaf was fully expanded for all accessions, the water application was terminated and the pots were transferred onto mesh table to accelerate soil drying.

During the drought treatment, the time-course changes in maximum quantum yield of photosystem II (F_v_/F_m_), stomatal conductance (g_s_) and green area were evaluated for every day up to 7th day after drought onset when all the plants were totally senescent. All the measurements were done with five pot replications during 4–6 h from the start of light period. At first, g_s_ was measured for a topmost fully expanded leaf using leaf porometer (SC-1, Decagon Devices, Pullman, WA, USA). Then we took a picture for a pot with scale from about 1 m just above using digital camera (μ730, Olympus, Tokyo, Japan). Subsequently, chlorophyll fluorescence was measured by Mini-PAM (Waltz Gmbh, Effeltrich, Germany) for the same leaf used for the g_s_ measurement. All the plants were dark adapted for 1 h prior to the measurement of minimum fluorescence (F_o_) under a weak modulating light. Thereafter, maximum fluorescence (F_m_) was determined by applying a saturation pulse. F_v_/F_m_ was calculated as F_v_/F_m_ = (F_m_ - F_o_)/F_m_ according to Maxwell and Johnson (2000).

The green area was estimated from the pictures using analysis software (ImageJ version1.46, National Institutes of Health; http://rsb.info.nih.gov/ij/). The relative ratio of green area was calculated as the value at drought onset was for 1.

We also determined the relative water content (RWC) on the 4th day after drought onset. The sampling was done for a topmost fully expanded leaf with three pot replications, and the RWC was determined according to Türkan et al. (2005).

## Results

### New *Vigna* accession found in a limestone rock mountain

**Figure 2:**
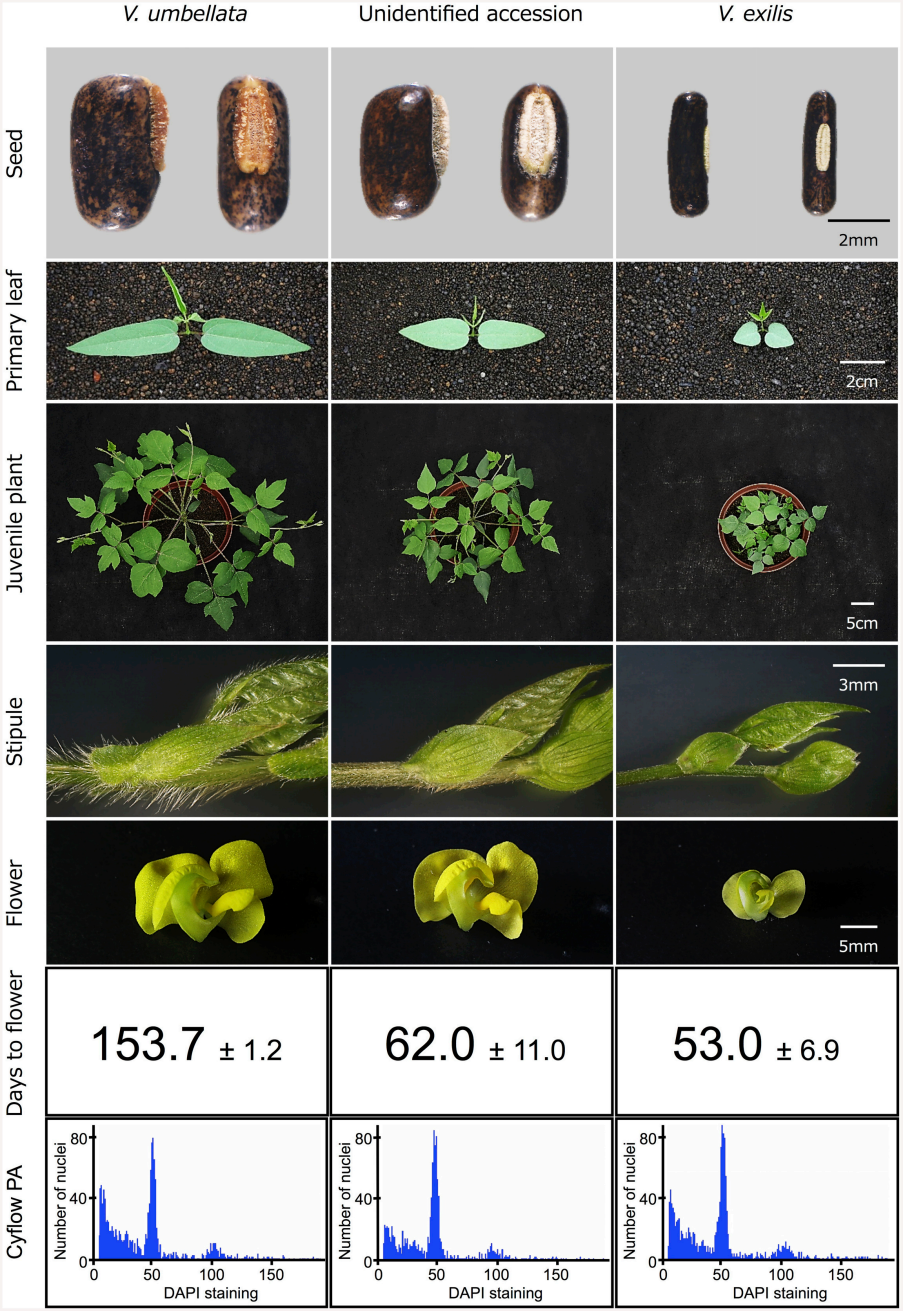
**Morphology of *V. umbellata*, *V. exilis* and the unidentified accession**.

In 1999, we explored Kanchanaburi district and Ratchaburi district in Thailand for *V. exilis*. We found, in addition to *V. exilis*, a *Vigna* population at the foot of a limestone rock mountain near Wat Rat Singkhon (Figure 1C). It looked similar to *V. umbellata*, but it was growing on limestone rocks where *V. umbellata* had never been found. We collected seeds of *V. exilis* (JP205884) and the *Vigna* plants (JP210644) that we recored as *V. umbellata*, since, the seed morphology was exactly the same as *V. umbellata* (see Figure 2; Table 1).

In 2012, we visited the same place and found the *Vigna* population formerly described as *V. umbellata* was vigorous and expanded up to middle of the limestone mountain, while the population of *V. exilis* had retarded (Figure 1C). We collected seeds of *V. exilis* (JP247172 and JP247173) and the *Vigna* plants (JP247174 and JP247175; Table 1).

In the second trip we suspected that the *Vigna* plants were hybrids of *V. exilis* and *V. umbellata*, because it was unlikely for *V. umbellata* to compete against *V. exilis* in a limestone environment. As such, we call the accessions of the *Vigna* plants as “unidentified” hereafter (Table 1).

### Morphology of the unidentified accessions

To test the hypothesis that the unidentified accessions are hybrids of *V. umbellata* and *V. exilis*, we cultivated *V. exilis* (JP205884), *V. umbellata* (JP207982), and the unidentified accession (JP210644) and observed the morphological characteristics in detail (Figure 2).

The seed of *V. umbellata* was rounded rectangle and had a large protruded hilum, while that of *V. exilis* was thin and had a small flat hilum. The primary leaves were sword-shaped in *V. umbellata* and heart-shaped in *V. exilis*. True leaves were mostly lobed in *V. umbellata*, but not in *V. exilis*. In growth type, *V. umbellata* vigorously extends both main stem and branches, while the main stem of *V. exilis* grew very slowly and tended to extend branches. *V. umbellata* developed brown, long, and dense trichomes on leaves and stems while *V. exilis* was almost hairless. Flowers of *V. umbellata* were large and had bright yellow color, while those of *V. exilis* had smaller, pale-yellow flower.

In the unidentified accession, the seed shape was almost impossible to be distinguished from *V. umbellata*. Other morphologies were intermediate of *V. umbellata* and *V. exilis*, though basically closer to *V. umbellata*. The first leaves were sword-shaped but short, the true leaves were slightly lobed, the growth type was similar to *V. umbellata* but slower, the trichomes were short, and the flowers looked like *V. umbellata* but a little smaller.

In addition, we found flowering time was greatly different between *V. umbellata* and *V. exilis*. To flower, it took 153.7 ± 1.2 days for *V. umbellata* after germination but only 53.0 ± 6.9 days for *V. exilis*. The flowering time was, contrary to other phenotypes, closer to *V. exilis* in the unidentified accession, where it took 62.0 ± 11.0 days to flower after germination.

### Phylogenetic relationship of the unidentified accessions and other species

To find out genetic relationship, we performed phylogenetic analyses on accessions of *V. umbellata, V. exilis*, and the unidentified accessions (Table 1), using sequences of the chloroplast *atpB-rbcL* spacer region, the rDNA-ITS region and the genotypes at 24 nuclear SSR sites (Tables 2–4, Supplementary Tables 2–4). We also included three accessions of *V. dalzelliana*, since *V. exilis* was once confused with this species (Tateishi and Maxted, 2002).

**Table 2:** **Polymorphic sites in *atpB-rbcL* spacer region**.

**Symbol**	**38**	**158**	**224**	**272**	**356**	**507**	**641**
umw1	T	G	T	G	–	A	–
umw2	T	G	T	G	–	A	–
umw3	T	G	T	G	–	A	–
umw4	T	G	T	G	–	A	T
uni1	T	G	T	G	–	A	–
uni2	T	G	T	G	–	A	–
uni3	T	G	T	G	–	A	–
exi1	G	T	T	T	T	C	–
exi2	G	T	T	T	T	C	–

**Table 3:** **Polymorphic sites in rDNA-ITS region**.

**Symbol**	**71**	**112**	**158**	**159**	**188**	**408**	**423**	**456**	**496**	**504**	**511**	**512**	**513**	**515**	**529**	**544**
umw1	C	A	G	C	C	C	G	T	A	A	C	A	A	G	Y^*^	W^*^
umw2	C	A	G	C	C	C	G	T	A	A	C	A	W^*^	G	Y^*^	W^*^
umw3	C	A	G	C	C	C	G	T	A	A	C	A	T	G	C	T
umw4	C	A	G	C	C	C	G	T	R^*^	A	C	A	A	G	T	A
uni1	G	G	G	C	S^*^	S^*^	G	K^*^	A	A	S^*^	R^*^	A	S^*^	T	A
uni2	G	G	G	C	S^*^	S^*^	G	K^*^	A	A	S^*^	R^*^	A	S^*^	T	A
uni3	G	G	G	C	S^*^	S^*^	G	K^*^	A	A	S^*^	R^*^	A	S^*^	T	A
exi1	G	G	A	T	G	G	A	G	A	G	G	G	A	C	T	A
exi2	G	G	A	T	G	G	A	G	A	G	G	G	A	C	T	A

* *R:G/A, Y:T/C, K:G/T, S:G/C, W:A/T*.

**Table 4:** **Genotypes of polymorphic SSR loci between *V. umbellata, V. exilis*, and the unidentified accessions**.

**SSR locus**	VES0019^*^	VES0093^*^	VES0427^*^	VES0478^*^	VES0678^*^	VES0749^*^	VES0987^*^	VES1258^*^	VES1469^*^
umw1	AA	AA	AA	AA	AA	AA	AA	AA	AA
umw2	AA	AA	AA	AA	AB	BB	AA	AA	AB
umw3	AA	AA	AA	AA	AA	BB	AA	AA	BB
umw4	BB	BB	AA	AA	AA	CC	AA	AA	AA
uni1	AA	BB	AA	AA	BB	EE	AA	UU	UU
uni2	AA	BB	AA	AA	BB	EE	AA	AA	UU
uni3	AA	BB	AA	AA	BB	EE	AA	AA	EE
exi1	EE	EE	EE	EE	EE	EE	EE	EE	EE
exi2	FF	EE	EE	EE	EE	EE	EE	EE	EE

* *A–C indicates alleles specific to V. umbellata, while E and F indicated those specific to V. exilis. U indicates an allele specific to the unidentified accessions*.

For the *atpB-rbcL* spacer region, 699 bp sequences were well-aligned to each other (Table 2, Supplementary Table 2). We detected six single nucleotide polymorphisms (SNPs) between *V. umbellata* and *V. exilis*, which were all the same as *V. umbellata* in all the unidentified accessions (Table 2, Supplementary Table 2). In addition, one intraspecific polymorphism was found in *V. umbellata* (a Thymine insertion at 671 nt in JP201676; Table 2, Supplementary Table 2). *V. dalzelliana* were greatly different from accessions of *V. umbellata, V. exilis* or the unidentified (Table 2, Supplementary Table 2).

For the rDNA-ITS region, 557 bp sequences were aligned to each other. We detected 12 SNP sites between *V. umbellata* and *V. exilis* (Table 3), and four SNP sites within *V. umbellata* (Supplementary Table 3). Of the 12 SNP sites in the unidentified accessions, two were *V. exilis*-type and four were *V. umbellata*-type, and six were double-signaled where we detected both *V. umbellata*-type and *V. exilis*-type SNPs within accessions (Table 3, Supplementary Table 3). Again, many polymorphisms specific to *V. dalzelliana* were found (Supplementary Table 3).

Of the 24 SSR sites tested, nine were polymorphic between accessions of *V. umbellata* and *V. exilis*. For example, at the locus of VES0478, where a 312 bp fragment was amplified in all *V. umbellata* accessions while a 309 bp fragment was amplified only in *V. exilis* accessions, all the three unidentified accessions were fixed with *V. umbellata*-type allele (Table 4, Supplementary Table 4). On the other hand, at the locus of VES0749, where the amplified fragment length was 226 bp in *V. exilis* and 208–223 bp in *V. umbellata*, the unidentified accessions were fixed with *V. exilis*-type allele (Table 4, Supplementary Table 4). In total, the unidentified accessions had seven SSR loci almost fixed with only *V. umbellata*-type alleles, one locus fixed with *V. exilis*-type, and one locus where an *V. exilis*-type allele and an allele specific to the unidentified accessions were present (Table 4, Supplementary Table 4).

**Figure 3:**
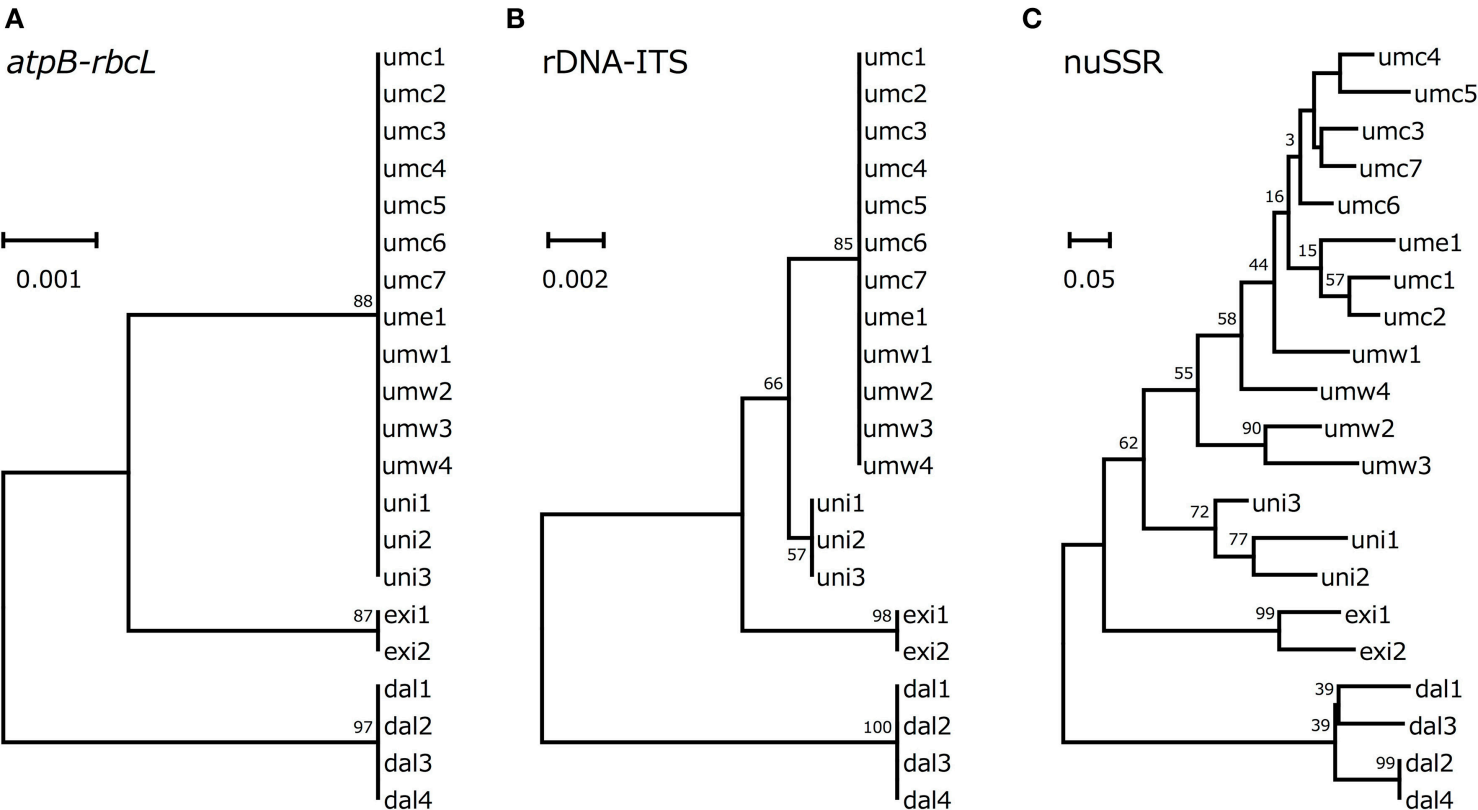
**Phylogenetic trees based on *atpB-rbcL* spacer sequences (A), rDNA-ITS (B), and genotypes of SSR loci (C)**.

According to the genotype data described above, we drew phylogenetic trees based on *atpB-rbcL* spacer sequences, rDNA-ITS sequences or SSR data. In all the three phylogenetic trees, *V. dalzelliana* accessions were the most divergent from *V. umbellata, V. exilis*, and the unidentified accessions (Figure 3). As expected, the unidentified accessions formed a single cluster with *V. umbellata* in the phylogenetic tree based on the *atpB-rbcL* spacer region (Figure 3A). In the phylogenetic trees based on nuclear genotypes, however, the unidentified accessions formed an independent cluster in between *V. umbellata* accessions and *V. exilis* accessions (Figures 3B,C). The phylogenetic tree based on SSR data indicated the unidentified accessions were closer to the wild accessions of *V. umbellata* than the cultivated accessions (Figure 3C).

### Ploidy in the unidentified accessions

Since most of the SSR loci were fixed and produced single bands in all the three unidentified accessions, they did not seem to have undergone polyploidization (Table 4, Supplementary Table 4). To further confirm that they have the same ploidy as *V. exilis* and *V. umbellata*, we examined ploidy level.

As a result, the amount of DNA in single nuclei was almost the same in *V. umbellata, V. exilis* and the unidentified accessions. In all the eleven accessions tested (see Table 1), the DAPI staining per nucleus showed the highest peak around the value of 50 (Figure 2).

### Cross compatibility

To test whether *V. exilis* or the unidentified accessions were truly cross-compatible with *V. umbellata*, we performed reciprocal crossing between *V. umbellata, V. exilis*, and an unidentified accession (Table 5).

**Table 5:** **Summary of artificial crossings**.

**Female**	**Male**	**No. of flower crossed**	**No. of pod obtained (length)**	**No. of seed obtained**
umw1	uni1	2	0	–
umw5	uni1	22	1 (6 cm)	8
umw1	exi3	10	0	–
umw5	exi3	27	1 (4 cm)	4
uni1	umw1, umw5	30	0	–
uni1	exi3	41	0	–
exi3	umw1, umw5	16	0	–
exi3	uni1	22	1 (1 cm)	0

As a result, F1 seeds were obtained only when *V. umbellata* served as a female parent (Table 5). The obtained F1 plants were confirmed by analyzing the SSR marker loci that were all heterozygous (Supplementary Table 5).

One case of successful pollination was also observed in a combination of *V. exilis* x the unidentified accession but no viable seeds were formed (Table 5).

### Alkaline tolerance

**Figure 4:**
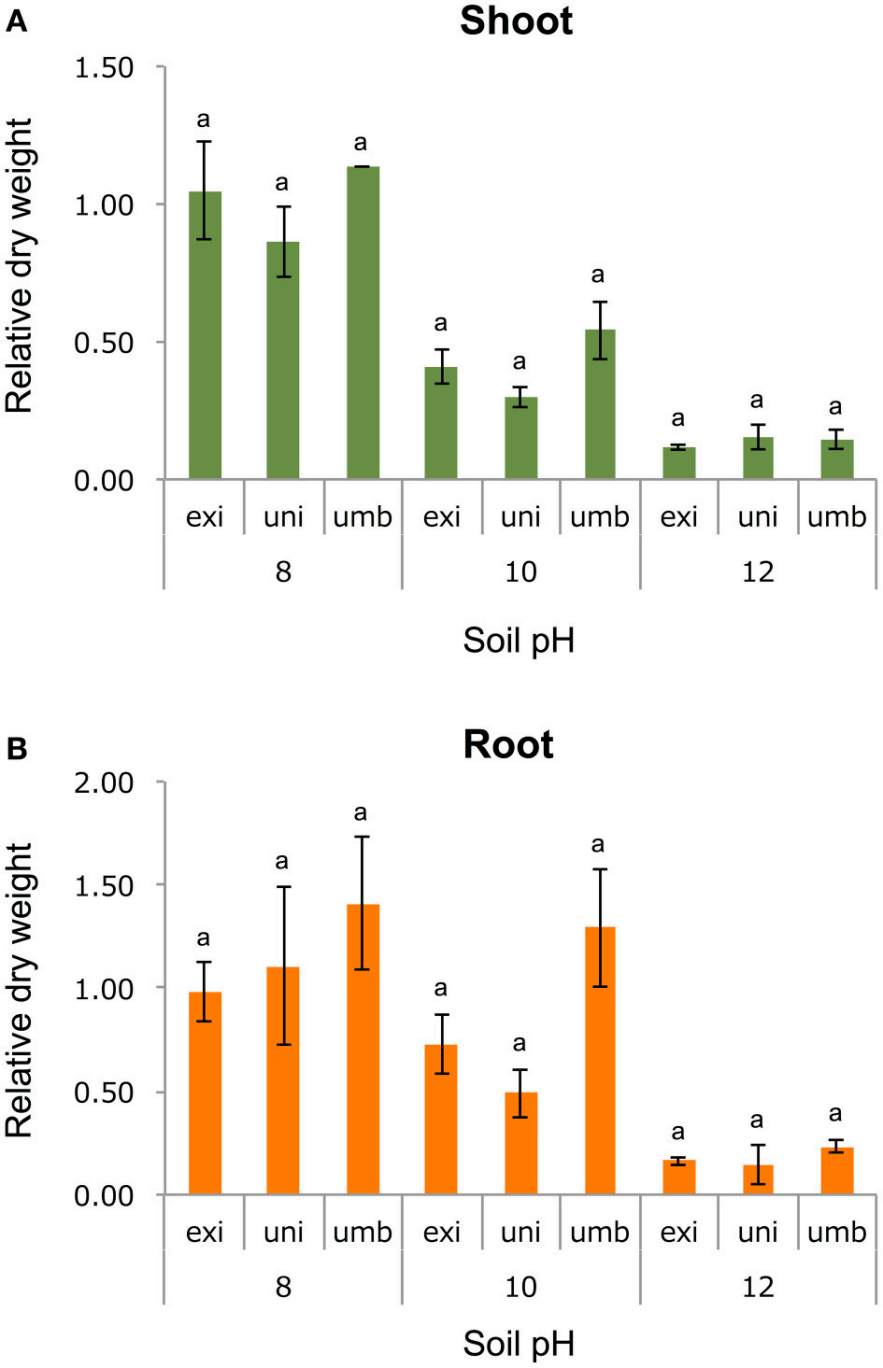
**The effect of high pH on *V. umbellata*, *V. exilis*, and the unidentified accession**. The y-axis indicates relative dry weight of the above-ground parts **(A)** and the roots **(B)** against control condition (pH6). No significant difference was observed between the three according to multiple comparison test by Turkey's range test.

To test one hypothesis that the adaptive trait of *V. exilis* and the unidentified accession to the limestone environment was alkaline tolerance, we cultivated the plants of *V. umbellata, V. exilis*, and an unidentified accession in conditions of pH6, pH8, pH10, or pH12 for 14 days. To evaluate the growth rate, we harvested and measured dry weight of above-ground parts and roots (Figure 4).

As a result, all accessions showed the best growth in pH6 or pH8. The dry weight of the whole plant bodies were 2.53 g (*SD* = 0.19), 0.30 g (*SD* = 0.08), and 1.18 g (*SD* = 0.01) for *V. umbellata, V. exilis*, and the unidentified accession, respectively.

Contrary to our expectations, however, *V. exilis* was not better in growth rate than *V. umbellata* in any pH conditions (Figure 4). In pH 10, *V. umbellata* showed the highest growth rate, although the difference was not significant. No significant difference in growth rate was observed between the three (Figure 4). The unidentified accession did not show significantly different growth rate, either (Figure 4).

### Drought tolerance

Since *V. exilis* lives in limestone mountains where the surface is covered with little soil, we suspected it would dry faster than soil-rich, lower lands. As such, we also tested the three accessions for drought tolerance.

**Figure 5:**
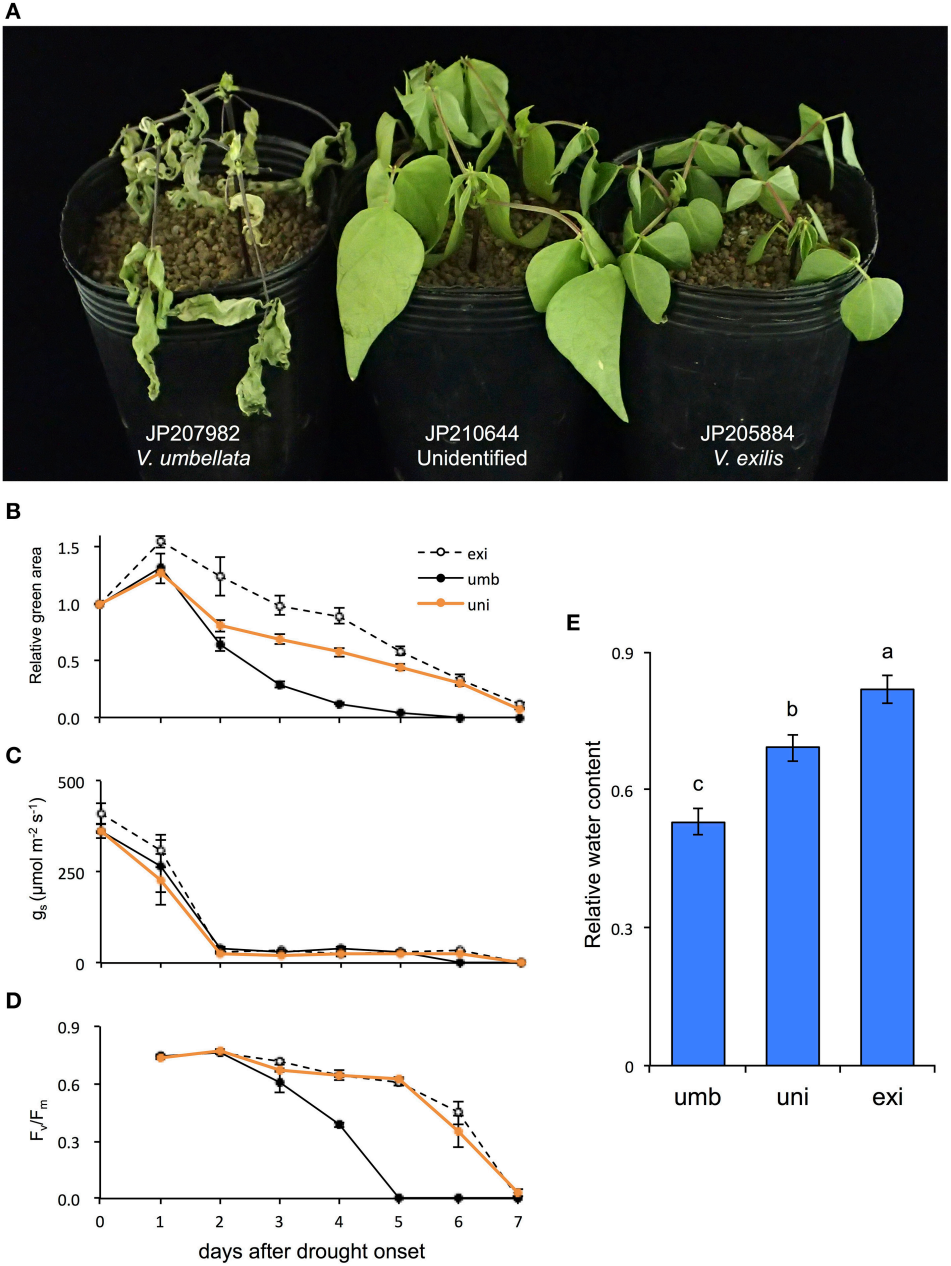
**The effect of drought stress on *V. umbellata*, *V. exilis*, and the unidentified accession**. Photo was taken on the 4th day after drought onset **(A)**. The effect of drought stress on relative green area **(B)**, stomatal conductance (g_s_) **(C)**, maximum quantum yield of photosystem II (F_v_/F_m_) **(D)**, and relative water content **(E)**. The values are presented as means ± standard error (SE); *n* = 5 for g_s_, relative green area and F_v_/F_m_; and *n* = 3 for relative water content. Bars with different letters are significantly different, denoted by *P* < 0.01 according to Turkey's range test.

As a result, the drought tolerance obviously differed among the accessions. On the 4th day *V. umbellata* was almost totally senescent, whereas *V. exilis* and the unidentified accession showed little symptoms of senescence (Figure 5A). Although the relative green areas decreased faster in the unidentified accession than in *V. exilis*, it decreased more rapidly in *V. umbellata* than both the others (Figure 5B). The half-life of the relative green areas was 3 days in *V. umbellata*, whereas those in *V. exilis* and the unidentified accession were 6 and 5 days, respectively (Figure 5B). All the accessions showed rapid decline of stomatal conductance [g_s_] which were decreased by 90% within two days after drought onset (Figure 5C). On the other hand, we observed great differences in the values of maximum quantum yields [F_v_/F_m_] (Figure 5D). The F_v_/F_m_ in *V. umbellata* started to decrease on the 3rd day and fell down to almost zero on the 5th day, whereas those in *V. exilis* and the unidentified accession showed little decrease during the same period. We also measured relative water content (RWC) on the 4th day and found it was significantly different from each other. RWC was the lowest in *V. umbellata*, the unidentified accession in the middle, and the highest in *V. exilis* (Figure 5E).

## Discussion

In this study, we have found a hybrid population derived from *V. umbellata* and *V. exilis*. Since the hybrid population is currently only identified in one location and seemed still expanding its habitat, we have possibly captured an early stage of homoploid hybrid speciation.

### The hybrid population in a limestone rock mountain

*V. exilis* lives only in steep-edged limestone hills in Southeast Asia, usually on outcrops of limestone rocks with its root growing into cracks of the rocks (Tomooka et al., 2002; Figure 1). In contrast, *V. umbellata* is found in various places such as mountain ranges and lowlands except the limestone environments. As such, it was surprising to find a population of *V. umbellata*-like plants in a limestone hill near Wat Rat Singkhon (Figure 1).

However, we found some *V. exilis*-like phenotypes such as less-lobed true leaves and shorter trichomes in the population (Figure 2), and because of its habitat, we suspected that it is a hybrid between *V. umbellata* and *V. exilis*. The following analysis using chloroplast DNA and nuclear DNA clearly revealed it is a hybrid (Figure 3, Tables 2–4, Supplementary Tables 2–4). The artificial pollination also produced viable seeds between *V. umbellata* and *V. exilis*, further confirming the probability of hybridization. The identical chloroplast *atpB-rbcL* spacer sequences between *V. umbellata* and the hybrid indicated the first step of the hybrid formation was a pollination of *V. exilis* pollen to *V. umbellata* (Table 2, Supplementary Table 2). In addition, according to the dominance of *V. umbellata*-type alleles at nuclear SSR loci, the hybrid had probably undergone at least one round of backcross by *V. umbellata* (Table 4, Figure 3), though we cannot rule out the possibility of biased segregation.

### The first homoploid hybrid in genus *Vigna*

In theory, hybrid speciation needs reproductive barriers such as alloploidy or geographical isolation (Rieseberg et al., 2003; Mao and Wang, 2011; Arnold et al., 2012). In genus *Vigna*, there is a case of allotetraploid species, *Vigna reflexo-pilosa* Hayata, which is derived from *Vigna hirtella* Ridley and *Vigna trinervia* (Heyne ex Wall.) Tateishi and Maxted (Chankaew et al., 2014b). Since, the hybrid population in 1999 and 2012 had little differences in SSR genotypes despite it is almost sympatric with its parental species *V. exilis*, we suspected that the hybrid is reproductively isolated from *V. exilis* by ploidy change.

However, the analysis on ploidy level undoubtedly confirmed that the hybrid retained the same ploidy level as its parents (Figure 2). Thus, this is the first case of homoploid hybrid in genus *Vigna*, and one of the few cases in the angiosperms (Paun et al., 2009).

Although we could not obtain F1 seeds between *V. exilis* and the hybrid, we do not consider we obtained enough data to claim they are cross-incompatible to each other. Since *V. umbellata* and *V. exilis* are cross compatible, it is difficult to suspect the hybrid has established a reproductive barrier against *V. exilis* without polyploidization. If so, however, how the hybrid population dominated bottom half of the limestone rock mountain with little gene flows at least for 13 years (Figure 1, Tables 4, 5, Supplementary Table 4)?

The flowering time difference can serve as a reproductive barrier for the parental species (Figure 2), however, it cannot explain the genetic stability of the hybrid population since there is a little flowering time difference between the two (Figure 2). As such, there might be an unknown mechanism to slow down gene flow from *V. exilis* to the hybrid (Table 4, Supplementary Table 4).

Another possibility is that the hybrid is so adaptive to the environment that the expansion of the hybrid population has outpaced the rate of gene flow from *V. exilis*. In any case, we need further studies with more markers and samples to elucidate the outcrossing rate between the hybrid and *V. exilis*. We should also keep observing whether the hybrid population further expand or not, and whether it would become ephemeral by repeated gene flow from *V. exilis*, if its expansion had slowed down or stopped.

### Adaptive traits for faster drying environment

Although the theory claims that acquisition of a transgressive phenotype is important for homoploid hybrids to colonize in ecological niches, there have been only a few studies which has successfully identified such “adaptive” traits in the hybrid species (Rieseberg et al., 2003; Mao and Wang, 2011; Arnold et al., 2012).

In this study, we expected it was easy to identify the key trait in the hybrid, since the hybrid and its parent *V. exilis* are living in limestone karsts, which mainly consists of calcium carbonate, but the other parent *V. umbellata* is not. Thus, we hypothesized that *V. exilis* is tolerant to alkaline stress, *V. umbellata* is sensitive, and the hybrid has acquired tolerance from *V. exilis*.

However, at least in the artificially alkalized condition, *V. umbellata* was not sensitive at all. *V. exilis, V. umbellata* and the hybrid showed the same or better performance in pH8 compared to control (pH6; Figure 4). As such, *V. umbellata* is also highly tolerant against high pH condition, and thus there should be other reasons for the mutually exclusive distribution between *V. exilis* and *V. umbellata*.

Our second hypothesis was drought tolerance. The limestone hills are covered with little soil, and thus rainwater quickly moves through the crevices into the ground. In contrast, the habitat of *V. umbellata* is soil-rich and is expected to dry slower.

As expected, the drought test clearly revealed that *V. exilis* is much more tolerant to drought stress than *V. umbellata*, and so was the hybrid (Figure 5). The higher survivability of *V. exilis* is probably due to avoidance of leaf dehydration because they retained higher RWC and the relative green area under the progressive drought conditions (Figures 5B,E). Although fast stomatal closure is an important response to prevent excessive water loss during drought (McDowell et al., 2008), no difference was observed in the timing and the degree of g_s_ decreases among the accessions (Figure 5C). As such, suppression of leaf dehydration might be due to other mechanisms such as thicker cuticles and osmotic adjustment (Sánchez et al., 1998; Kerstiens, 2006). In any case, the dehydration avoidance enabled *V. exilis* and the hybrid to maintain their photosystem activity under the prolonged drought (Figure 5D). All these results strongly indicated that the drought tolerance provided *V. exilis* and the hybrid with higher fitness to the faster drying conditions.

The difference in flowering time might also have important meaning (Figure 2). In the faster drying condition, plants have shorter time to grow and thus need to reproduce before available water runs out. On the other hand, in the slower drying conditions plants with longer vegetative growth phase can grow bigger and produce more offspring (Cohen, 1976; Kozlowski, 1992; Franks et al., 2007). As such, the earlier flowering in *V. exilis* and the hybrid is probably due to selection from the former conditions, whereas the delayed flowering in *V. umbellata* is a selected trait for the latter.

Although we are not currently sure about the contribution of *V. umbellata* to the fitness of the hybrid, the hybrid is more vigorous than *V. exilis* and that is why the hybrid has dominated the bottom half of the limestone rock mountain (Figures 1, 2).

### The value of the hybrid as a genetic resource

Last but not least, we should note that the hybrid has a great potential as a genetic resource. The hybrid is not only as tolerant as *V. exilis* to drought stress, but it has much larger plant biomass than *V. exilis*. As such, the hybrid indicated compatibility of drought tolerance and large biomass. The wide crossability of *V. umbellata* would make the hybrid even more valuable (Gupta et al., 2002; Tomooka et al., 2002). We have previously shown that *V. umbellata* is cross compatible with most species belonging to section Angulares, including azuki bean (*V. anguraris*) and rice bean (*V. umbellata*; Tomooka et al., 2002). Moreover, it can even produce viable seeds when crossed with mungbean (*V. radiata*) and black gram (*Vigna mungo* (L.) Hepper), which belongs to section Ceratotropis (Gupta et al., 2002). As such, we expect the hybrid is also crossable to these crop species for improvement in drought tolerance.

## Author contributions

YT, KI, and KN designed experiments and wrote the manuscript. KK and CM analyzed data. PS, KI, and NT helped draft the manuscript.

### Conflict of interest statement

The authors declare that the research was conducted in the absence of any commercial or financial relationships that could be construed as a potential conflict of interest.
