# Supplementary material for: A Homoploid Hybrid Between Wild Vigna Species Found in a Limestone Karst
Source: Front Plant Sci. 2015 Dec 1;6:1050. doi: 10.3389/fpls.2015.01050 (PMC4664699; doi:10.3389/fpls.2015.01050)
Supplement: Supplementary file 1 [file Table1.PDF]

Supplementary Material

A homoploid hybrid between wild *Vigna* species found in a limestone karst

Yu Takahashi, Kohtaro Iseki, Kumiko Kitazawa, Chiaki Muto, Prakit Somta, Kenji Irie, Ken Naito*, Norihiko Tomooka

* Correspondence: Ken Naito: knaito@affrc.go.jp

Supplementary Table 1. Accession numbers of the *atpB-rbcL* and rDNA-ITS sequences obtained in this study.

Symbol	NIAS Accession No.	DDBJ Accession No	
		atpB-rbcL	rDNA-ITS
uni1	JP210644	LC064324	LC064303
uni2	JP247174	LC064325	LC064304
uni3	JP247175	LC064326	LC064305
umw1	JP207982	LC064327	LC064306
umw2	JP210639	LC064328	LC064307
umw3	JP210642	LC064329	LC064308
umw4	JP210676	LC064330	LC064309
umc1	JP212059	LC064331	LC064310
umc2	JP217439	LC064332	LC064311
umc3	JP223027	LC064333	LC064312
umc4	JP223046	LC064334	LC064313
umc5	JP225373	LC064335	LC064314
umc6	JP239864	LC064336	LC064315
umc7	JP227454	LC064337	LC064316
ume1	JP210647	LC064338	LC064317
exi1	JP247172	LC064339	LC064318
exi2	JP247173	LC064340	LC064319
dal1	JP210812	LC064341	LC064320
dal2	JP210813	LC064342	LC064321
dal3	JP210815	LC064343	LC064322
dal4	JP210816	LC064344	LC064323
