# Supplementary material for: A Homoploid Hybrid Between Wild Vigna Species Found in a Limestone Karst
Source: Front Plant Sci. 2015 Dec 1;6:1050. doi: 10.3389/fpls.2015.01050 (PMC4664699; doi:10.3389/fpls.2015.01050)
Supplement: Supplementary file 2 [file Table2.PDF]

Supplementary Material

A homoploid hybrid between wild *Vigna* species found in a limestone karst

Yu Takahashi, Kohtaro Iseki, Kumiko Kitazawa, Chiaki Muto, Prakit Somta, Kenji Irie, Ken Naito*, Norihiko Tomooka

* Correspondence: Ken Naito: knaito@affrc.go.jp

Supplementary Table 2. Polymorphic sites in *atpB-rbcL* spacer region.

Symbol	68	188	242	243	244	245	246	247	248	254	302	317	321	386	537	538	553	671	682
exi1	G	T	-	-	-	-	-	-	-	T	T	-	A	T	C	A	A	-	T
exi2	G	T	-	-	-	-	-	-	-	T	T	-	A	T	C	A	A	-	T
uni1	T	G	-	-	-	-	-	-	-	T	G	-	A	-	A	A	A	-	T
uni2	T	G	-	-	-	-	-	-	-	T	G	-	A	-	A	A	A	-	T
uni3	T	G	-	-	-	-	-	-	-	T	G	-	A	-	A	A	A	-	T
umw1	T	G	-	-	-	-	-	-	-	T	G	-	A	-	A	A	A	-	T
umw2	T	G	-	-	-	-	-	-	-	T	G	-	A	-	A	A	A	-	T
umw3	T	G	-	-	-	-	-	-	-	T	G	-	A	-	A	A	A	-	T
umw4	T	G	-	-	-	-	-	-	-	T	G	-	A	-	A	A	A	T	T
ume1	T	G	-	-	-	-	-	-	-	T	G	-	A	-	A	A	A	-	T
umw1	T	G	-	-	-	-	-	-	-	T	G	-	A	-	A	A	A	-	T
umw2	T	G	-	-	-	-	-	-	-	T	G	-	A	-	A	A	A	-	T
umw3	T	G	-	-	-	-	-	-	-	T	G	-	A	-	A	A	A	-	T
umw4	T	G	-	-	-	-	-	-	-	T	G	-	A	-	A	A	A	-	T
umw5	T	G	-	-	-	-	-	-	-	T	G	-	A	-	A	A	A	-	T
umw6	T	G	-	-	-	-	-	-	-	T	G	-	A	-	A	A	A	-	T
umw7	T	G	-	-	-	-	-	-	-	T	G	-	A	-	A	A	A	-	T
dal2	G	G	T	A	T	C	A	T	T	A	G	T	G	-	C	C	-	-	A
dal3	G	G	T	A	T	C	A	T	T	A	G	T	G	-	C	C	-	-	A
dal4	G	G	T	A	T	C	A	T	T	A	G	T	G	-	C	C	-	-	A
dal5	G	G	T	A	T	C	A	T	T	A	G	T	G	-	C	C	-	-	A
