# Supplementary material for: A Homoploid Hybrid Between Wild Vigna Species Found in a Limestone Karst
Source: Front Plant Sci. 2015 Dec 1;6:1050. doi: 10.3389/fpls.2015.01050 (PMC4664699; doi:10.3389/fpls.2015.01050)
Supplement: Supplementary file 3 [file Table3.PDF]

Supplementary Material

A homoploid hybrid between wild *Vigna* species found in a limestone karst

Yu Takahashi, Kohtaro Iseki, Kumiko Kitazawa, Chiaki Muto, Prakit Somta, Kenji Irie, Ken Naito*, Norihiko Tomooka

* Correspondence: Ken Naito: knaito@affrc.go.jp

Supplementary Table 3. Polymorphic sites in rDNA-ITS spacer region.

Symbol	52	67	70	79	81	83	84	85	89	90	92	100	121	136	140	157	163	168
exi1	T	A	G	C	G	T	C	C	G	G	C	T	G	G	C	A	C	T
exi2	T	A	G	C	G	T	C	C	G	G	C	T	G	G	C	A	C	T
uni1	T	A	G	C	S*	T	Y*	S*	G	G	T	T	G	G	M*	A	C	T
uni2	T	A	G	C	S*	T	Y*	S*	G	G	T	T	G	G	M*	A	C	T
uni3	T	A	G	C	S*	T	Y*	S*	G	G	T	T	G	G	M*	A	C	T
umw1	W*	R*	G	C	C	T	T	G	G	G	T	T	G	G	A	A	C	T
umw2	W*	R*	G	C	C	W*	T	G	G	G	T	T	G	G	A	A	C	T
umw3	A	G	G	C	C	A	T	G	G	G	T	T	G	G	A	A	C	T
umw4	T	A	G	C	C	T	T	G	G	G	T	Y*	G	G	A	A	C	T
ume1	W*	A	G	C	C	T	T	G	G	G	T	Y*	G	G	A	A	C	T
umc1	T	A	G	C	C	T	T	G	G	G	T	Y*	G	G	A	A	C	T
umc2	T	A	G	C	C	T	T	G	G	G	T	Y*	G	G	A	A	C	T
umc3	T	A	G	C	C	T	T	G	G	G	T	C	G	G	A	A	C	T
umc4	T	A	G	C	C	T	T	G	G	G	T	Y*	G	G	A	A	C	T
umc5	T	A	G	C	C	T	T	G	G	G	T	Y*	G	G	A	A	C	T
umc6	T	A	G	C	C	T	T	G	G	G	T	Y*	G	G	A	A	C	T
umc7	W*	A	G	C	C	T	T	G	G	G	T	Y*	G	G	A	A	C	T
dal2	T	A	G	T	G	T	C	A	A	G	T	T	A	R*	C	A	G	G
dal3	T	A	R*	T	G	T	C	A	A	S*	T	T	A	G	C	R*	G	G
dal4	T	A	G	T	G	T	C	A	A	S*	T	T	A	G	C	R*	G	G
dal5	T	A	G	T	G	T	C	A	A	G	T	T	A	R*	C	A	G	G

*R: G/A, Y: T/C, M: A/C, K: G/T, S: G/C, W: A/T

Supplementary Table 3. Continued.

Symbol	173	188	189	193	212	213	408	437	438	461	463	484	521	522	525
exi1	T	C	T	C	C	G	C	A	T	A	G	C	G	G	C
exi2	T	C	T	C	C	G	C	A	T	A	G	C	G	G	C
uni1	C	S*	T	C	C	G	S*	G	C	A	G	C	G	G	C
uni2	C	S*	T	C	C	G	S*	G	C	A	G	C	G	G	C
uni3	C	S*	T	C	C	G	S*	G	C	A	G	C	G	G	C
umw1	C	G	T	C	C	G	G	G	C	A	G	T	R*	K*	G
umw2	C	G	T	C	C	G	G	G	C	A	G	T	G	G	G
umw3	C	G	T	C	C	G	G	G	C	A	G	T	G	G	G
umw4	C	G	T	C	C	G	G	G	C	A	G	T	G	G	G
ume1	C	G	T	C	C	G	G	G	C	A	G	T	G	G	G
umc1	C	G	T	C	C	G	G	G	C	A	G	T	G	G	G
umc2	C	G	T	C	C	G	G	G	C	A	G	T	G	G	G
umc3	C	G	T	C	C	G	G	G	C	A	G	T	G	G	G
umc4	C	G	T	C	C	G	G	G	C	A	G	T	G	G	G
umc5	C	G	T	C	C	G	G	G	C	A	G	T	G	G	G
umc6	C	G	T	C	C	G	G	G	C	A	G	T	G	G	G
umc7	C	G	T	C	C	G	G	G	C	A	G	T	G	G	G
dal2	G	G	G	T	G	T	C	G	T	G	T	T	G	G	C
dal3	G	G	G	T	G	T	C	G	T	G	T	T	G	G	C
dal4	G	G	G	T	G	T	C	G	T	G	T	T	G	G	C
dal5	G	G	G	T	G	T	C	G	T	G	T	T	G	G	C

*R: G/A, Y: T/C, M: A/C, K: G/T, S: G/C, W: A/T
