# Supplementary material for: A Homoploid Hybrid Between Wild Vigna Species Found in a Limestone Karst
Source: Front Plant Sci. 2015 Dec 1;6:1050. doi: 10.3389/fpls.2015.01050 (PMC4664699; doi:10.3389/fpls.2015.01050)
Supplement: Supplementary file 4 [file Table4.PDF]

Supplementary Material

A homoploid hybrid between wild *Vigna* species found in a limestone karst

Yu Takahashi, Kohtaro Iseki, Kumiko Kitazawa, Chiaki Muto, Prakit Somta, Kenji Irie, Ken Naito*, Norihiko Tomooka

* Correspondence: Ken Naito: knaito@affrc.go.jp

Supplementary Table 4. The amplified fragment lengths at the SSR loci

Symbol	VES0019		VES0093		VES0116		VES0202		VES0204		VES0335		VES0427		VES0478		VES0670		VES0678		VES0749		VES0762	
exi1	282	282	183	183	286	286	128	128	341	341	273	273	335	335	309	309	105	105	315	315	226	226	253	253
exi2	291	291	183	183	286	286	128	128	341	341	270	270	335	335	309	309	105	105	315	315	226	226	253	253
uxi1	276	276	192	192	289	289	128	128	327	327	273	273	334	334	312	312	93	103	322	322	226	226	244	244
uxi2	276	276	192	192	289	289	128	128	326	326	273	273	334	334	312	312	103	103	322	322	226	226	250	250
uxi3	276	276	192	192	286	289	128	128	327	327	273	273	334	334	312	312	103	103	322	322	226	226	253	253
umw1	276	276	193	193	286	289	129	129	333	333	273	273	334	334	312	312	105	105	318	318	208	208	253	253
umw2	272	276	193	193	286	289	128	128	341	374	270	273	334	334	312	312	105	105	318	322	218	218	250	250
umw3	276	276	193	193	286	286	128	128	341	341	270	270	334	334	312	312	105	105	318	318	218	218	253	253
umw4	272	272	192	192	286	286	128	128	333	333	273	273	334	334	312	312	105	105	318	318	223	223	253	253
ume1	282	282	193	193	286	286	129	129	350	350	273	273	334	334	312	312	105	105	318	318	223	223	253	253
umc1	276	276	193	193	286	286	129	129	333	333	272	272	334	334	312	312	105	105	318	318	223	223	253	253
umc2	276	276	193	193	286	286	129	129	350	350	273	273	334	334	312	312	105	105	318	318	223	223	253	253
umc3	285	275	192	192	286	286	129	129	333	333	273	273	334	334	312	312	105	105	318	318	208	208	253	253
umc4	282	282	193	193	286	286	129	129	333	333	273	273	334	334	312	312	105	105	318	318	208	208	253	253
umc5	282	282	192	192	289	289	129	129	333	333	273	273	334	334	312	312	105	105	318	318	208	208	253	253
umc6	276	285	193	193	286	286	129	129	333	333	273	273	334	334	312	312	105	105	318	318	208	208	253	253
umc7	276	276	192	192	286	286	129	129	333	333	273	273	334	334	312	312	105	105	318	318	208	208	253	253
dal1	269	269	194	194	286	286	128	128	314	314	273	273	334	334	317	317	93	100	322	322	211	211	237	237
dal2	272	272	194	194	286	286	128	128	314	314	273	273	334	334	313	313	103	103	322	322	211	211	244	244
dal3	269	269	194	194	286	286	128	128	314	314	273	273	334	334	317	317	103	103	322	322	211	211	244	244
dal4	272	272	194	194	286	286	128	128	314	314	273	273	334	334	313	313	103	103	322	322	211	211	244	244

Supplementary Table 4. Continued.

Symbol	VES0769		VES0987		VES1023		VES1029		VES1067		VES1085		VES1258		VES1263		VES1271		VES1310		VES1469		VES777	
exi1	324	324	314	314	149	149	119	119	487	487	262	262	403	403	318	318	318	318	309	309	130	130	167	167
exi2	324	324	314	314	149	149	119	119	486	486	262	262	403	403	319	319	318	318	309	309	130	130	167	167
uxi1	324	324	313	313	149	149	119	119	487	487	262	262	401	401	319	319	318	318	309	309	144	144	167	167
uxi2	324	324	313	313	149	152	119	119	486	486	262	262	400	400	319	319	318	318	309	309	144	144	167	167
uxi3	322	324	313	313	149	152	119	119	487	487	262	262	400	400	319	319	318	318	309	309	130	130	167	167
umw1	321	324	313	313	152	152	119	119	487	487	259	259	400	400	319	319	318	318	303	309	139	139	167	167
umw2	324	324	313	313	149	152	119	119	487	487	259	262	400	400	319	319	318	318	303	303	139	150	167	167
umw3	322	322	313	313	149	149	119	119	487	487	259	259	400	400	319	319	318	318	303	303	150	150	167	167
umw4	324	324	313	313	152	152	119	119	487	487	262	262	400	400	319	319	318	318	309	309	139	139	167	167
ume1	322	322	313	313	152	152	119	119	487	487	262	262	400	400	319	319	318	318	303	303	139	139	167	167
umc1	322	322	313	313	152	152	119	119	486	486	262	262	400	400	319	319	318	318	306	306	139	139	167	167
umc2	322	322	313	313	152	152	119	119	486	486	262	262	400	400	319	319	318	318	306	306	139	139	167	167
umc3	322	322	313	313	152	152	119	119	487	487	262	262	400	400	319	319	318	318	306	306	139	139	167	167
umc4	322	322	313	313	152	152	119	119	486	486	262	262	400	400	319	319	318	318	306	306	139	139	167	167
umc5	323	323	313	313	152	152	119	119	486	486	262	262	400	400	319	319	318	318	306	306	139	139	167	167
umc6	324	324	313	313	152	152	119	119	487	487	262	262	400	400	319	319	318	318	306	306	139	139	167	167
umc7	323	323	313	313	152	152	119	119	487	487	262	262	400	400	319	319	318	318	306	306	139	139	167	167
dal1	325	325	310	310	145	145	121	121	487	487	262	262	403	403	318	318	314	314	306	306	133	133	176	176
dal2	325	325	310	310	145	145	121	121	487	487	262	262	403	403	318	318	314	314	306	306	133	133	176	176
dal3	325	325	310	310	139	139	121	121	487	487	262	262	403	403	318	318	314	314	306	306	142	142	176	176
dal4	325	325	310	310	145	145	121	121	487	487	262	262	403	403	318	318	314	314	306	306	133	133	176	176
