# Supplementary material for: A Homoploid Hybrid Between Wild Vigna Species Found in a Limestone Karst
Source: Front Plant Sci. 2015 Dec 1;6:1050. doi: 10.3389/fpls.2015.01050 (PMC4664699; doi:10.3389/fpls.2015.01050)
Supplement: Supplementary file 5 [file Table5.PDF]

Supplementary Material

A homoploid hybrid between wild *Vigna* species found in a limestone karst

Yu Takahashi, Kohtaro Iseki, Kumiko Kitazawa, Chiaki Muto, Prakit Somta, Kenji Irie, Ken Naito[*], Norihiko Tomooka

* Correspondence: Ken Naito: knaito@affrc.go.jp

Supplementary Table 5. Genotypes of SSR loci in the F1 plants and parental lines.

Species	Status	JP No.	VES19	VES93_2	VES749	VES1023	VES1258	VES1263
V. umbellata	Wild	251332	261 261	192 192	222 222	151 151	387 387	311 311
V. umbellata	Wild	251332	261 261	192 192	222 222	151 151	387 387	311 311
V. umbellata x *V. exilis*	F1	-	261 267	182 192	214 222	148 151	387 390	311 317
V. umbellata x *V. exilis*	F1	-	261 267	182 192	214 222	148 151	387 390	311 317
V. exilis	Wild	205884	267 267	182 182	214 214	148 148	390 390	317 317
V. exilis	Wild	205884	267 267	182 182	214 214	148 148	390 390	317 317
